# Maternal response to environmental unpredictability

**DOI:** 10.1002/ece3.1723

**Published:** 2015-10-05

**Authors:** Miguel Barbosa, Isabel Lopes, Catia Venâncio, Maria João Janeiro, Michael Blair Morrisey, Amadeu M.V.M. Soares

**Affiliations:** ^1^ CESAMDepartamento de BiologiaUniversidade de AveiroCampus de Santiago3810AveiroPortugal; ^2^ Scottish Oceans InstituteUniversity of St AndrewsSt AndrewsFifeKY16 8LBUK; ^3^ School of BiologyUniversity of St AndrewsSt AndrewsFifeKY16 8LBUK; ^4^ Programa de Pós‐Graduação em Produção VegetalUniversidade Federal do TocantinsCampus de Gurupi77402‐970GurupiBrazil

**Keywords:** Fitness, jack of all trades, maternal Investment, trans‐generational effects, unpredictability, variance

## Abstract

Mothers are expected to use environmental cues to modify maternal investment to optimize their fitness. However, when the environment varies unpredictably, cues may not be an accurate proxy of future conditions. Under such circumstances, selection favors a diversifying maternal investment strategy. While there is evidence that the environment is becoming more uncertain, the extent to which mothers are able to respond to this unpredictability is generally unknown. In this study, we test the hypothesis that *Daphnia magna* increase the variance in maternal investment in response to unpredictable variation in temperature consistent with global change predictions. We detected significant variability across temperature treatments in brood size, neonate size at birth, and time between broods. The estimated variability within‐brood size was higher (albeit not statistically significant) in mothers reared in unpredictable temperature conditions. We also detected a cross‐generational effect with the temperature history of mothers modulating the phenotypic response of F1's. Notably, our results diverged from the prediction that increased variability poses a greater risk to organisms than changes in mean temperature. Increased unpredictability in temperature had negligible effects on fitness‐correlated traits. Mothers in the unpredictable treatment, survived as long, and produced as many F1's during lifetime as those produced in the most fecund treatment. Further, increased unpredictability in temperature did not affect the probability of survival of F1's. Collectively, we provide evidence that daphnia respond effectively to thermal unpredictability. But rather than increasing the variance in maternal investment, daphnia respond to uncertainty by being a jack of all temperatures, master of none. Importantly, our study highlights the essential need to examine changes in variances rather than merely on means, when investigating maternal responses.

## Introduction

The role of environmental conditions in shaping maternal investment is unequivocal (Mousseau and Fox 1998). In many systems, mothers use environmental cues to predict the environmental conditions of their offspring, and adjust their maternal allocation in ways that optimize their fitness. Environments have a natural pattern of change (e.g., seasonality), and when this change is predictable, directional maternal allocation strategies are likely to evolve (Stearns 1992; Simons 2011). There is, however, evidence that overall environmental conditions are becoming more unpredictable (Morice et al. 2012; Mora et al. 2013), and if so, directional maternal allocation strategies may be maladaptive. Instead, under unpredictable conditions, selection predicts the evolution of a maternal strategy that promotes phenotypic variability (Slatkin 1974; Beaumont et al. 2009; Crean and Marshall 2009; Starrfelt and Kokko 2012; Rajon et al. 2014). Despite strong support for the evolution of plastic reproductive strategies in response to unpredictability (Nussey et al. 2005; Fischer et al. 2011), empirical evidence that mothers increase the variance in maternal allocation in response to environmental unpredictability remains scarce (Barbosa et al. 2014; Singh et al. 2015). Here we address this gap and, test the hypothesis that environmental unpredictability leads to increased variance in maternal allocation.

A consequence of environmental unpredictability is that some traits are optimal at one time but disadvantageous at another (Grant and Grant 2002). There is, therefore, high variation in fitness when the environment is unpredictable. One way, in which, mothers can reduce this variation and shield against total reproductive failure, is by promoting variability in reproductive investment. For example, by increasing the variability in reproductive allocation within or between broods, mothers ensure that the fitness costs of producing a brood under nonoptimal conditions are minimized (Cohen 1966; Marshall and Uller 2007).

Global temperature is becoming more variable (Morice et al. 2012; Mora et al. 2013; Karl et al. 2015), which is worryingly expected to pose a greater adaptive pressure to organisms than a mean increase in temperature (Vasseur et al. 2014). There is, therefore, the challenge to identify potential mechanisms of adaptation to increased unpredictability in the variability in temperature. Increasing the variance in maternal allocation has been shown to allow organisms to respond successfully to mean changes in environmental conditions (Beaumont et al. 2009). Whether or not mothers increase the variance in reproductive allocation in response to unpredictability in temperature, as theoretically expected (Cohen 1966; Mousseau and Fox 1998), remains, however, poorly understood (Gremer and Venable 2014). Here, we address this gap and tested the hypothesis that the water flea *Daphnia magna* increases the variance in reproductive allocation in response to increased variability in temperature. Trait variability plays a vital role in evolutionary adaptation (Barbosa et al. 2010; González‐Suárez et al. 2015), for this reason in this study, maternal responses were quantified in terms of their variance rather than focusing on changes in the mean.

*Daphnia magna* responses to environmental heterogeneity were tested by quantifying the variance in brood size, time between broods and length at birth, three temperature‐dependent (Cooper et al. 2005) and maternal‐correlated fitness traits (Bernado 1996), over two complete generations. Daphnia are dominant organisms in ephemeral habitats. Their adaptive success in thriving under unpredictable environments is partiality due to their facility to adjust maternal investment. Numerous studies show that daphnia adjust the quality and number of neonates, their size, and disease resistance, in response to changes in temperature, food availability, and predation risk (Lynch and Ennis 1983; Glazier 1992; Barbosa et al. 2014; Garbutt et al. 2014).

There is strong evidence that the evolution of diversifying strategies are more likely to occur at intermediate levels of grain scale, in which individuals go through different environments at random temporal scales throughout life (Levins 1968; Venail et al. 2011). In order to accommodate this, the variance within mothers was examined for three reproductive traits, across four temperature treatments: low, mean, high (coarse grain), and unpredictable (variable – fine grain), over two complete generations (F1 and F2). Further, by quantifying the variance within mothers across generations, we use a stronger test which allows us to better partition the coarse‐grained (intergenerational) and fine‐grained (intragenerational) scales in maternal responses (Schoeppner and Relyea 2009).

Numerous studies described a direct link between maternal conditions and offspring response to environmental stressors (Mitchell and Read 2005; Garbutt et al. 2014). It is then predicted that offspring fitness will be influenced by the conditions experienced by mothers. While measuring fitness is intrinsically difficult (Hunt and Hodgson 2010), it is recognized that total number of offspring produced through life is an accurate proxy for maternal fitness (Hunt and Hodgson 2010; Barbosa et al. 2012). Further, for many organisms, the probability of survival and fecundity are two fitness‐correlated traits shown to be influenced by the maternal rearing temperature (Mousseau and Dingle 1991; Tregenza et al. 2003). The fitness consequences of increased unpredictability in temperature were then tested by recording the probability of F1 survival and fecundity at both F0 and F1 (i.e., number of F2 produced) in a full factorial design of maternal and offspring environmental treatments.

## Methods

### Source generation

All F0 individuals (*N*_F0_ = 20) used in this study were 3rd brood neonates generated from *D. magna* clone F (Schoeppner and Relyea 2009). All source individuals (individuals used only to generate F0's used in our experimental test) were raised at a constant temperature of 20°C in a 16‐/8‐h light: dark photoperiod in ASTM (American Society for Testing Materials) and fed with green algae *Pseudokirchneriella subcapitata*, at a concentration of 3.0 x 10^5^ cells mL^−1^. Because all source individuals were kept under the same temperature, photoperiod, and feeding regimes, we ensure that differences in F0 responses during their experimental test were not caused by differences in the conditions of the source individuals.

### F0 generation

Immediately after birth, 20 F0 individuals were randomly allocated between four temperature treatments, low (*N* = 5, 15°C), mean (*N* = 5, 20°C), high (*N* = 5, 25°C), and unpredictable (*N* = 5, Δ 15 to 25°C).

We decided to set the lower and upper temperature limited at 15°C and 25°C, respectively, because *Dapnhia magna* reproductive performance and probability of survival is significantly affected around the boundary of this interval (Mitchell and Read 2005). The temperature in the unpredictable treatment varied stochastically on a daily basis. We were interested in quantifying maternal responses to meaningful variation and temporal pace of change in temperature, as forecasted by climate change (Burton‐Chellew et al. 2008). With this in mind, the temperature in the unpredictable temperature treatment varied according to two subgroups – 00:00 to 08:00/ 18:00 to 24:00 (dawn‐morning/late afternoon) and from 08:00 to 18:00 (morning and afternoon). In the dawn‐morning/ late afternoon, temperature fluctuated unpredictably between 15°C and 20°C. In the morning and afternoon group, it varied between 20°C and 25°C. The mean temperature in the unpredictable treatment was 19.8°C. The mean temperatures in the unpredictable and in the mean treatment were similar, thus any effect observed in the unpredictable treatment could be unambiguously attributed to differences in predictability rather than on different mean temperatures.

Each F0 individual was placed in a 20‐mL glass vial using a 3‐mL plastic pipette and then randomly allocated to a temperature treatment in a Binder incubator (Binder Bs28). There was one incubator per temperature treatment. In the unpredictable temperature treatment, the incubator controller was set with a maximum, minimum, and daily variation in temperature. All F0 individuals remained in their temperature treatments from birth to death.

### Neonate generation (F1)

F1 neonates were checked every day (*N*_F1 _= 4799). The number of days between broods, the number of neonates per brood, and individual length of each neonate were recorded at every brood produced by each individual F0 for the entire life. After birth, each individual F1 was placed in a culture cell plate using a 3‐mL plastic pipette and its photograph taken for measuring body length (from the tip of the head to the start of caudal spine) to the nearest millimeter using ImageJ software. Following that each F1 was allocated to a glass vial and randomly assigned to either their maternal treatment or to one of the other temperature treatments. By relocating the F1 generation into the maternal temperature treatment, we increased the power of replication at the clonal and experimental level (*N*_F1 _= 4799). Any effect of temperature could, therefore, be unambiguously detected. All F1's remained in their treatment for their entire life and time between broods, and the number and length at birth of each F2 produced were recorded (*N*_F2_ = 134663).

Both F0 and F1 were fed daily with green algae *Pseudokirchneriella subcapitata,* and their medium was changed every two days. F0 and F1 individuals remained in their temperature treatment until they died. The experiment finished when the last F1 individual died.

### Statistics

We used linear mixed models (LMM) to test the hypothesis that mothers increase the variance in their maternal allocation in response to unpredictable variations in temperature. Our general strategy was to fit models that assumed that within‐mother (for response variable brood size and time between broods) and within‐brood (for response variable neonate length at birth) variance differed among temperature treatments (H1), and to compare these models to ones that assumed common within‐mother/brood variance across treatments (H0). This procedure was used for both F0 and F1. The exception was that, for computational reasons, we could not estimate the within‐brood variance in F2 neonate length at birth. Each response variable (i.e., brood size, neonate length at birth and time between broods) was modeled separately in both F0 and F1.

We were interested in investigating maternal responses in terms of their variance. Therefore, models with heterogeneous variance, in particular with different variance among temperature treatments, were fit to obtain within‐mother/brood variance estimates for each treatment (Pinheiro and Bates 2000). We then used likelihood ratio tests (LRT) to compare these models to models assuming the same variance for all treatments and therefore to test whether variance is different among temperature treatments. The assumed null distribution of twice the difference in log likelihoods between nested models is *chi‐square* distributed, with degrees of freedom equal to the difference in the number of parameters between H1 and H0. Models accounting for temporal correlation, which included autoregressive correlation structures, were also fit in order to investigate whether variance estimates would differ significantly from the models without such structure. As that was not the case, the most parsimonious models were kept. Significant differences in variance between treatments can occur because of the existence of outliers in some treatments. A sensitivity analysis was carried out to investigate the leverage of outliers, defined as observations corresponding to normalized residuals greater than the 0.975 quantile of the standard normal distribution.

The behavior of each response variable is expected to differ markedly through time (Dieter E. Ecology, Epidemiology, and Evolution of Parasitism in Daphnia Bethesda (MD): National Library of Medicine (US), National Center for Biotechnology, 2005). We therefore fitted fixed effects structures for each trait that would model the average response of the population to age and treatment, so that differences among treatments on average effects would not be mistaken for differences among treatments in within‐mother variances. There is evidence that brood size increases after sexual maturation and decreases after 2 months, whereas time between broods is constant through life (Dieter E. Ecology, Epidemiology, and Evolution of Parasitism in Daphnia Bethesda (MD): National Library of Medicine (US), National Center for Biotechnology, 2005). Although daphnia grow indeterminably throughout life, growth rate slows down with time. Therefore, the fixed effect structure for the three models developed for F0, one for each response variable (y), included treatment, age, a quadratic term for age, and an interaction between age and treatment. For F1 models, we adopted a simplified model to avoid overparameterization and improve interpretability. The fixed structure for F1 included, F0 treatment, F1 treatment, and the interaction between them. Finally, diagnostic plots revealed that time between broods does not follow a normal distribution, showing a heavy right tail. Time between broods was therefore log‐transformed. All results for time between broods refer to the transformed variable.

The effect of temperature on lifetime reproductive success was examined using a linear mixed model (LMM). We first compared the total number of F1 neonates between F0 temperature treatments. In order to test whether increased variation in temperature leads to greater fitness in the long term, we also compared the total number of F2 produced during lifetime via F0 temperature treatments. We used the same fixed and random effects and residual variance structures as used for the above models.

Finally, we investigated the effect of increased variation in temperature on the probability of survival in F0 mothers using a Cox proportional hazard model. Further, to test for potential adaptive consequences of increase unpredictability in temperature, we compared survival curves estimates between F1's from different maternal treatments and reared under the same maternal condition or under a different one, while controlling for the between F0 and between F1 variation.

All analyses were performed in R (Team RDC 2013), using packages nlme (Pinheiro and Bates 2000) and coxme (Therneau 2015).

## Results

**Figure 1:**
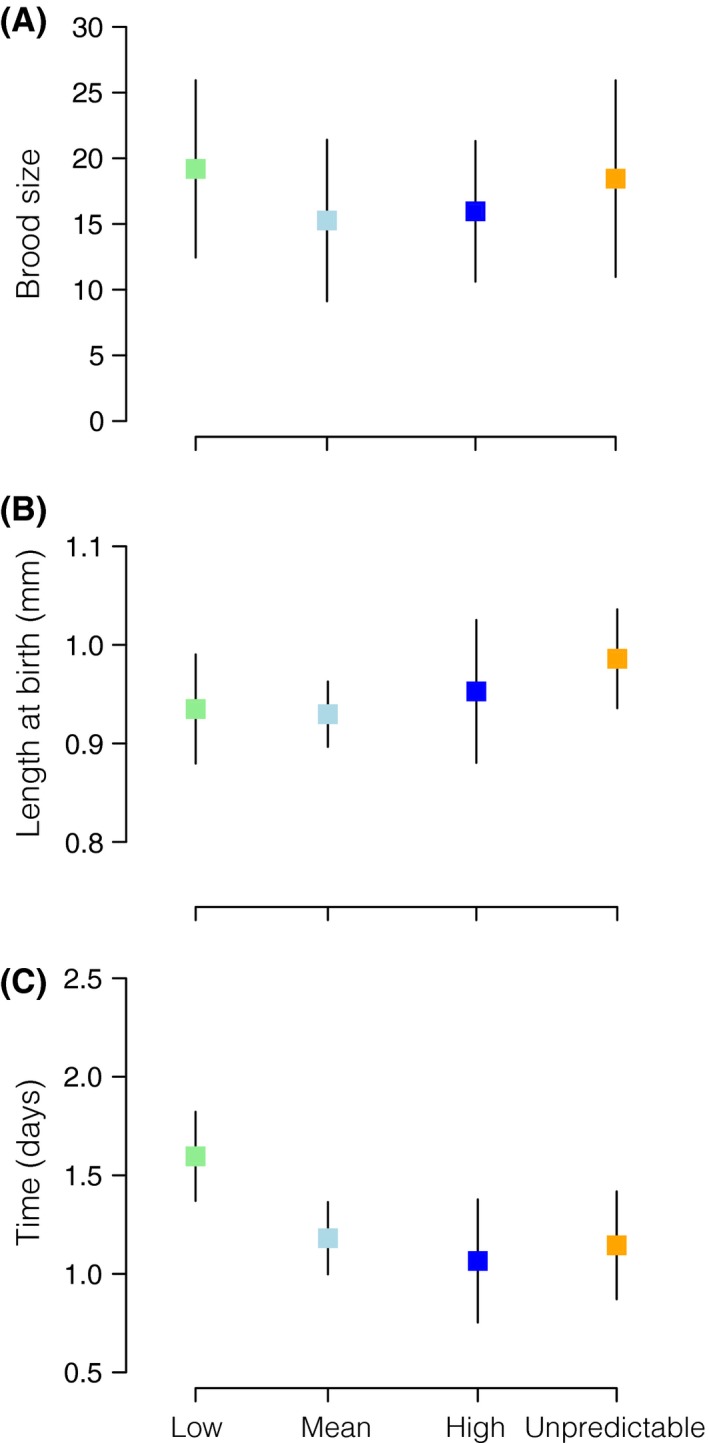
Coefficients of within‐F0 mother/brood estimates of standard deviation between treatments for (A) brood size, (B) length at birth, and (C) time between broods. Error bars denote standard deviation.

**Figure 2:**
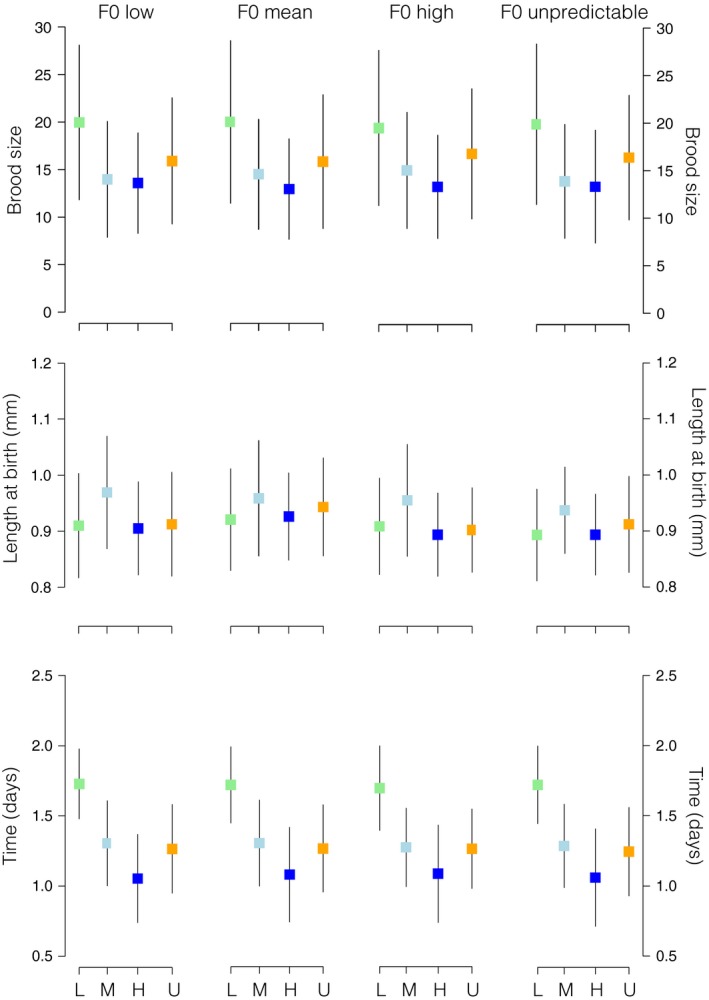
Coefficients of within‐F1 estimates of standard deviation for brood size, length at birth and time between broods, for F1 neonates reared under low (light green), mean (light blue), high (dark blue) or unpredictable (orange) temperature treatments. F1 are nested within their mother's temperature treatment. Error bars denote standard deviation.

We detected significant differences among temperature treatments in within‐F0 mother variability for brood size (*P *=* *0.027; Table 1). The estimated within‐F0 mother standard deviation of brood size was greatest in the unpredictable environment and lowest at high temperatures (SD: low = 6.49, mean = 6.16, high = 5.32, unpredictable = 7.46; Figs. 1, S1). Mean brood size, as a function of F0 age, was significantly different between treatments (*P *=* *0.011; Table 3). We also detected a significant heterogeneity among F1 treatments for brood size (*P *=* *0.009; Table 2, Figs. 2, S1). With both F0 and F1, temperature treatments contributing significantly for the heterogeneity in F1 brood size (Table 2, Fig. 2). The estimated within‐mother variability in F1 brood size was, on average, greatest in the low temperature treatment (S1).

**Table 1:** Comparison of the models with different within‐F0 mother/brood variances among temperature treatments to the models with only one residual variance for all treatments, using likelihood ratio test. *P* values considered significant for *P *< 0.05

Model designation	Model				df	LL	2Δlnl	*P*
	Response variable	Fixed effects	Random effects	Variance structure				
Heteroscedastic within F0	Brood size	F0 treatment * age + age^2^	F0 ID	F0 treatment	14	−951.8		
Homoscedastic		F0 treatment * age + age^2^	F0 ID		11	−956.4	9.109	0.027
Heteroscedastic within F0	Neonate length at birth	F0 treatment * age + age^2^	F0 ID + Brood	F0 treatment	15	2775.6		
Homoscedastic		F0 treatment * age + age^2^	F0 ID + Brood		12	2659.7	231.8	< 0.001
Heteroscedastic within F0	Time between broods	F0 treatment * age + age^2^	F0 ID	F0 treatment	14	−9.982		
Homoscedastic		F0 treatment * age + age^2^	F0 ID		11	−18.16	16.36	<0.001

**Table 2:** Comparison of the models with different within F0 and F1 variances among temperature treatments to the models with only one residual variance for all treatments, using likelihood ratio test. *P* values considered significant for *P *< 0.05

Model designation	Model				df	LL	2Δlnl	*P*
	Response variable	Fixed effects	Random effects	Variance structure				
Heteroscedastic within F0 and F1	Brood size	F0 treatment * F1 treatment	F0 ID/F1 ID	F0 treatment * F1 treatment	34	−2799		
Homoscedastic		F0 treatment * F1 treatment	F0 ID/F1 ID		19	−2816	351.1	<0.001
Heteroscedastic within F0		F0 treatment * F1 treatment	F0 ID/F1 ID	F0 treatment	22	−2816	349.4	<0.001
Heteroscedastic within F1		F0 treatment * F1 treatment	F0 ID/F1 ID	F1 treatment	22	−2798	26.23	0.009
Heteroscedastic within F0 and F1	Neonate length at birth	F0 treatment * F1 treatment	F0 ID/F1 ID	F0 treatment * F1 treatment	34	5629		
Homoscedastic		F0 treatment * F1 treatment	F0 ID/F1 ID		19	5562	1356	<0.001
Heteroscedastic within F0		F0 treatment * F1 treatment	F0 ID/F1 ID	F0 treatment	22	5582	947.4	<0.001
Heteroscedastic within F1		F0 treatment * F1 treatment	F0 ID/F1 ID	F1 treatment	22	5594	702.5	<0.001
Heteroscedastic within F0 and F1	Time between broods	F0 treatment * F1 treatment	F0 ID/F1 ID	F0 treatment * F1 treatment	34	−1902		
Homoscedastic		F0 treatment * F1 treatment	F0 ID/F1 ID		19	−1947	90.45	<0.001
Heteroscedastic within F0		F0 treatment * F1 treatment	F0 ID/F1 ID		22	−1944	84.12	<0.001
Heteroscedastic within F1		F0 treatment * F1 treatment	F0 ID/F1 ID		22	−1912	20.63	0.055

We also identified significant differences among treatments in the amount of within‐mother/brood variability for neonate length at birth in F0 (*P *<* *0.001; Table 1). F0 mothers maintained at constant high temperature had the greatest estimated within‐brood standard deviation in neonate length (SD; low = 0.06, mean = 0.03, high = 0.07, unpredictable = 0.05; Figs. 1, S1). There were significant differences in length of neonates produced as a function of age (*P *<* *0.001; Table 3). As for F1 brood size, we also detected a significant interaction between F0 treatment and F1 treatment on the amount of within‐brood variability for F2 neonate length at birth (*P *<* *0.001; Table 2, Figs. 2, S1). We found greater variability in length at birth of F2 via F0 and F1 reared in the mean temperature treatment.

**Table 3:** Fixed effect structure for each response variable. For better inference of average slope versus curvature parameters, estimates were mean‐centered by age. *P* values considered significant for *P *< 0.05

Response variable	Fixed effects	Estimate	df	SE	*T*‐value	*P*
Brood size	Low	18.90	16	1.081	17.51	<0.001
	Mean	15.42	16	0.761	20.27	<0.001
	High	15.90	16	0.674	23.61	<0.001
	Unpredictable	18.49	16	0.925	19.99	<0.001
	Age	0.172	266	0.054	3.209	0.002
	Age^2^	−0.002	266	0.001	−1.633	0.104
	Mean * Age	−0.261	266	0.064	−4.058	<0.001
	High * Age	−0.311	266	0.069	−4.539	<0.001
	Unpredictable * Age	−0.176	266	0.069	−2.566	0.011
Neonate length at birth	Low	0.931	16	0.013	69.47	<0.001
	Mean	0.984	16	0.013	76.87	<0.001
	High	0.936	16	0.019	50.48	<0.001
	Unpredictable	0.934	16	0.014	67.14	<0.001
	Age	0.001	99	0.001	1.359	0.177
	Age^2^	0000	99	0000	−5.022	<0.001
	Mean * Age	0.001	99	0.001	1.237	0.219
	High * Age	0.003	99	0.001	2.825	0.006
	Unpredictable * Age	0.001	99	0.001	0.621	0.536
Time between broods	Low	1.597	16	0.037	42.84	<0.001
	Mean	1.667	16	0.022	53.09	<0.001
	High	1.024	16	0.029	35.46	<0.001
	Unpredictable	1.136	16	0.033	34.94	<0.001
	Age	−0.002	266	0.002	−1.333	0.184
	Age^2^	0000	266	0000	2.042	0.042
	Mean * Age	0.007	266	0.002	3.253	<0.001
	High * Age	0.011	266	0.003	3.642	<0.001
	Unpredictable * Age	0.004	266	0.002	1.642	0.102

The estimated amount of within‐F0 mother variability in time between broods was higher in F0 mothers in the high temperature treatment (SD: low = 0.22, mean = 0.18, high = 0.31, unpredictable = 0.27; Figs. 1, S1) (*P *<* *0.001; Table 1). Time between broods as a function of age was also significantly different between maternal conditions (*P *<* *0.001; Table 3). As with number of neonates, time between broods, as a function of age, was highest in F0 mothers allocated to the low temperature treatment. We also detected a marginally significant variability within F1 mothers in time between broods (*P *=* *0.05; Table 2, Figs. 2, S1). Variability in time between broods was greater within F1 mothers in the high temperature treatment that had been produced by F0 mothers that were also reared in the high temperature treatment.

**Figure 3:**
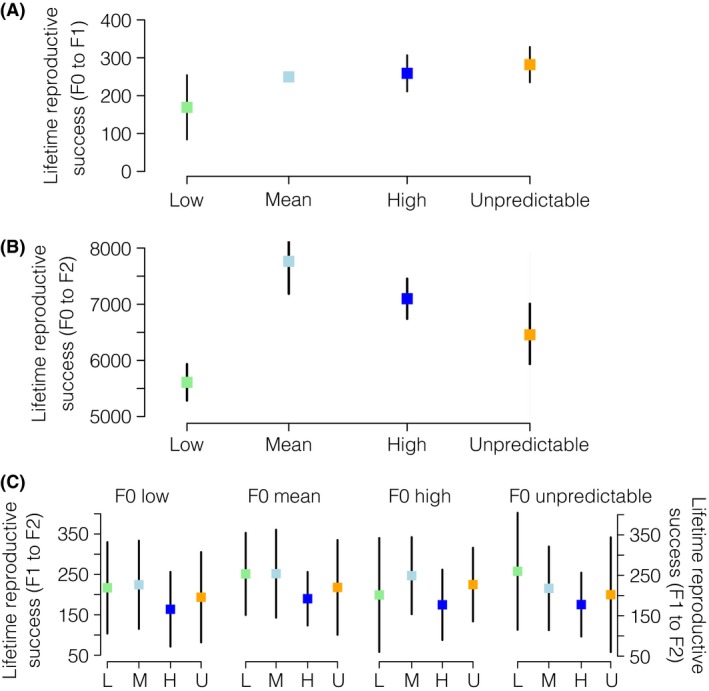
Mean total number of neonates produced during lifetime by F0 and F1 reared under low (light green), mean (light blue), high (blue), or unpredictable (orange) temperature regimes. Mean total number of F1 neonates produced during lifetime by F0 (A), mean total number of F2 neonates produced during lifetime via F0 (B) or via F1 neonates (C). L, M, H, and U indicate low, mean, high, and unpredictable treatments, respectively. Whiskers indicate the estimate standard errors of the model. Total lifetime reproductive success of F0 and F1's shown on S2.

F0 maternal treatment had no significant effect on lifetime reproductive success (*P *=* *0.192). However, F0 mothers reared under unpredictable temperature produced more F1 neonates than F0 mothers allocated to the other temperature treatments (Figs. 3, S2). There were significantly more F2 neonates produced during lifetime via F0 mothers that were allocated to the mean, high, and unpredictable temperature treatments than those produced via F0 mothers in the low treatment (*P *=* *0.008; Figs. 3, S2). There was, however, a significant effect of F1 treatment on the number of F2 neonates produced during lifetime (*P *<* *0.001; Fig. 3). F1's allocated to the mean temperature treatment produced more F2 neonates than the other temperature treatments (Figs. 3, S2).

There were no differences in the probability of survival between F0 treatments (*χ* ^2 ^= 1.71, *P *=* *0.634). Nevertheless, F0's reared under unpredictable temperature conditions survived the longest (mean (±SD) = 66 (16.6) days). Also, F0 treatment had no effect on the probability of F1 survival (*χ* ^2 ^= 4.39, *P *=* *0.221). F1 neonates produced via F0 that were reared under unpredictable temperature treatment lived on average 65 (SD ± 33) days. Only F1 neonates produced by F0 allocated to the mean temperature treatment lived longer (mean (±SD) = 70 (28.9) days). Further, regardless the F0 temperature treatment, F1 neonate probability of survival was not significantly different between them when allocated to different temperatures (*χ* ^2 ^= 1.94, *p*_low _= 0.58; *χ* ^2 ^= 4.27, *p*_mean _= 0.23; *χ* ^2 ^= 2.99, *p*_high _= 0.39; *χ* ^2 ^= 4.71, *p*_unpredictable _= 0.19).

## Discussion

Our lifetime analysis of variation in maternal reproductive investment detected no consistent effect of environmental unpredictability to generate increased variance in reproductive traits. While the estimates of variance in brood size were higher under unpredictable conditions, they were not statistically different from two other treatments (mean and high temperature). Moreover, estimates of within‐mother/brood variance in F1 neonate length at birth and time between broods were greater within‐broods allocated to the high temperature treatment, rather than the unpredictable treatment. Curiously, our results also indicate that the temperature conditions of the F0 generation interact with the temperature conditions of the F1 generation to create increased variance in F1 brood size (i.e., number of F2's), F2 neonate length at birth and time between broods. Notably, we failed to attest the suggestion that increased unpredictability in temperature poses a greater fitness costs than shifts in mean temperature. Instead, increased unpredictability in temperature was shown to have negligible effects in lifetime reproductive success and on the probability of survival. F0 mothers allocated to the unpredictable treatment produced as many F1 and F2 neonates than mothers allocated to either the mean or high temperature treatments. Further, fecundity in unpredictable temperature treatment did not come at the costs of increased mortality, as increased variability in temperature did not affect the probability of survival of both F1 and F2.

Changes in temperature generate metabolic costs, which determine the allocation of resources into reproductive and/or somatic growth (van Noordwijk and de Jong 1986). Given the limiting amount of energy available for reproduction, mothers may respond to unpredictable conditions by maximizing fitness over shorter lifetime (i.e., producing always larger broods sizes than the optimal brood size number (Seger and Brockmann 1987)). An alternative possibility is one in which mother's alternate reproductive investment according to metabolic costs of current conditions. Here, mothers would increase fecundity under optimal temperature conditions, but reduce reproductive investment when the metabolic costs of temperature increases. Our results are partially in agreement with this latter hypothesis as brood sizes varied greatly when temperature was unpredictable than when temperature was constantly high. Under fluctuating temperature conditions, the optimal temperature for each cellular process is likely to be encountered throughout life. On the other hand, when temperature remains constantly below or above optimal, some critical process may stop. There is evidence that daphnia are susceptible to increases in mean temperature, with sharper declines in fitness when temperature is above optimal conditions (Martin and Huey 2008). The smaller variation in brood size at high, but not low, temperature observed in our results is likely to be an adaptive response to the greater energetic costs of high temperature.

While our failure to detect an effect of unpredictable temperature on length at birth is interesting, it is not entirely unexpected (see (McKee 1997) for similar results). Under benign predation conditions, selective mechanisms such as length‐dependent predation are excluded. Studies with daphnia have reported diminishing gains in fitness with increasing offspring length (Tessier and Consolatti 1989; Boersma 1997), which may denote a weak selection on offspring length. Instead, in the absence of length‐selective mortality, selection favors mothers to invest in fecundity rather than size (Morrongiello et al. 2012). Examining the combine effects of increase variability in temperature under different predation regimes on daphnia length at birth should shed some noteworthy insights into the selection on this trait in daphnia.

Many species respond to unpredictability in environmental conditions by increasing the variation in time between broods, and this has been consistently characterized as maternal bet hedging (Simons 2011; Gremer and Venable 2014). For daphnia, time between broods has been shown to be very variable (Dieter E. Ecology, Epidemiology, and Evolution of Parasitism in Daphnia Bethesda (MD): National Library of Medicine (US), National Center for Biotechnology, 2005; Bradley et al. 1993). Our estimates of within‐mother standard deviation in time between broods were greater for mothers reared at both high and unpredictable temperatures, than those reared under low and mean temperatures. This lends support to previous studies. Our result is that we add that variation in time between broods is trigger by both an increased in mean temperature as well as an increased in the variability (unpredictability) in temperature.

Both F0 and F1 treatment conditions contributed to changes in phenotypic response different temperature conditions. This result is consistent with recent evidence that maternal effects have trans‐generational consequences (Coleman et al. 2014; Olof and McNamara 2015). Nevertheless, neonates in the unpredictable temperature treatment that were produced by mothers also in the unpredictable temperature treatment did not have greater estimates of within‐variance than neonates from the other temperature treatments. It has also been suggested that offspring produced from mothers experiencing strong directional selection will cope better when faced with the same maternal conditions but significantly worse if conditions change (Kelly et al. 2011). While F0 treatment did not affect F1 probability of survival, F1's in the high and unpredictable temperature treatment had a decreased in lifetime reproductive success relative to their mothers. The fitness cost observed on the second generation may indicate that long‐term exposure to extreme high or unpredictable temperatures has an impact on the reproductive success. The potential fitness costs described for F1 fecundity strongly suggest that studies that focus on a single generation may fail to observe any negative effects of unpredictable variation in temperature.

Any maternal response to a given environmental context is only adaptive if it translates into greater maternal fitness (Marshall and Uller 2007; Burgess and Marshall 2011). F0 reared at unpredictable temperatures produced more neonates during lifetime (albeit not statistically significant from mean and high temperature treatment) than mothers in the low temperature treatment. For some insect species, suboptimal temperature leads to physiological dysfunctions, which may cause a reduction in fecundity (Levie et al. 2005). This could provide with an explanation for the lower fecundity at low temperature treatment. The greater fecundity in the unpredictable treatment was not achieved at the expense of lower probability of survival. Instead, there were no differences in the probability of survival in both F0 and F1 of unpredictable temperature treatment. We could not, therefore, confirm that unpredictable changes in temperature pose a greater threat than shifts in mean temperature (Rahmstorf and Coumou 2011; Vasseur et al. 2014). By contrast, we provide strong evidence that daphnia are able to maximize fitness even when conditions are unpredictable.

A recent study suggest that asynchrony among different traits can stabilize populations through a portfolio effect (Moore et al. 2014). Analogous to this idea is the concept of jack of all trades, in which organisms are on average better at everything but not excellent at any (Levins 1968). Our results indicate that for all traits studied, and specifically for fitness‐correlated traits, individuals reared under unpredictable temperature always performed as good as individuals reared under the optimal temperature treatment. In order words, individuals reared under unpredictable temperatures produced as many F1 neonates as the most fecund temperature treatment at no costs of probability of survival. It is then possible that the success of daphnia in coping with thermal unpredictably results from being a “jack of all temperatures, master of none” strategy, rather than through any evolutionary advantages of increased variance in maternal reproductive investment. By being “good on average” in all traits at the sacrifice of maximal performance, daphnia can cope more efficiently with unpredictable variation in temperature than with constant decrease/increase in temperatures (i.e., low or high). Our results, therefore, support the classical principle of allocation (Levins 1968) as an adaptive mechanism to allow species to cope with future uncertainty.

## Conflict of Interest

None declared.

## Supporting information


**Table S1.** Means and standard deviation from the fitted model of estimated amount of within‐variability in F1 brood size, F2 neonate length at birth and time between gestation.
**Table S2.** The total lifetime number of neonates produced by F0s and F1s in each temperature treatment. Means and standard deviation are presented (mean ± SD).
