# Supplementary material for: Maternal response to environmental unpredictability
Source: Ecol Evol. 2015 Oct 5;5(20):4567–77. doi: 10.1002/ece3.1723 (PMC4670057; doi:10.1002/ece3.1723)
Supplement: Supplementary file 1 — Table S1. Means and standard deviation from the fitted model of estimated amount of within‐variability in F1 brood size, F2 neonate length at birth and time between gestation. Table S2. The total lifetime number of neonates produced by F0s and F1s in each temperature treatment. Means and standard deviation are presented (mean ± SD). [file ECE3-5-4567-s001.docx]

| Trait | | F0 treatment | F1 treatment | Mean | Standard deviation |
| --- | --- | --- | --- | --- | --- |
| Brood size | | Low | Low | 19.95 | 8.127 |
|  |  | Low | Mean | 13.96 | 6.095 |
|  |  | Low | High | 13.57 | 5.284 |
|  |  | Low | Unpredictable | 15.89 | 6.629 |
|  |  |  |  |  |  |
|  |  | Mean | Low | 20.01 | 8.551 |
|  |  | Mean | Mean | 14.50 | 5.780 |
|  |  | Mean | High | 12.94 | 5.285 |
|  |  | Mean | Unpredictable | 15.83 | 7.025 |
|  |  |  |  |  |  |
|  |  | High | Low | 19.48 | 8.154 |
|  |  | High | Mean | 15.02 | 6.095 |
|  |  | High | High | 13.30 | 5.431 |
|  |  | High | Unpredictable | 16.77 | 6.845 |
|  |  |  |  |  |  |
| Neonate length at birth | | Low | Low | 0.909 | 0.093 |
|  |  | Low | Mean | 0.969 | 0.100 |
|  |  | Low | High | 0.904 | 0.083 |
|  |  | Low | Unpredictable | 0.912 | 0.092 |
|  |  |  |  |  |  |
|  |  | Mean | Low | 0.920 | 0.090 |
|  |  | Mean | Mean | 0.958 | 0.103 |
|  |  | Mean | High | 0.926 | 0.077 |
|  |  | Mean | Unpredictable | 0.943 | 0.087 |
|  |  |  |  |  |  |
|  |  | High | Low | 0.908 | 0.086 |
|  |  | High | Mean | 0.954 | 0.099 |
|  |  | High | High | 0.893 | 0.074 |
|  |  | High | Unpredictable | 0.901 | 0.075 |
|  |  |  |  |  |  |
|  |  | Unpredictable | Low | 0.893 | 0.081 |
|  |  | Unpredictable | Mean | 0.937 | 0.076 |
|  |  | Unpredictable | High | 0.893 | 0.072 |
|  |  | Unpredictable | Unpredictable | 0.911 | 0.085 |

S1 – Means and standard deviation from the fitted model of estimated amount of within-variability in F1 brood size, F2 neonate length at birth and time between gestation.

S1 (continued)

| Trait | | F0 treatment | F1 treatment | Mean | Standard deviation |
| --- | --- | --- | --- | --- | --- |
| Time between broods | | Low | Low | 1.727 | 0.249 |
|  |  | Low | Mean | 1.305 | 0.302 |
|  |  | Low | High | 1.053 | 0.313 |
|  |  | Low | Unpredictable | 1.264 | 0.315 |
|  |  |  |  |  |  |
|  |  | Mean | Low | 1.716 | 0.271 |
|  |  | Mean | Mean | 1.300 | 0.306 |
|  |  | Mean | High | 1.075 | 0.337 |
|  |  | Mean | Unpredictable | 1.262 | 0.310 |
|  |  |  |  |  |  |
|  |  | High | Low | 1.691 | 0.301 |
|  |  | High | Mean | 1.268 | 0.279 |
|  |  | High | High | 1.081 | 0.346 |
|  |  | High | Unpredictable | 1.259 | 0.283 |
|  |  |  |  |  |  |
|  |  | Unpredictable | Low | 1.720 | 0.276 |
|  |  | Unpredictable | Mean | 1.285 | 0.296 |
|  |  | Unpredictable | High | 1.058 | 0.347 |
|  |  | Unpredictable | Unpredictable | 1.243 | 0.315 |
|  |  |  |  |  |  |

S2 – The total lifetime number of neonates produced by F0s and F1s in each temperature treatment. Means and standard deviation are presented (mean ± SD).

| F0 treatment | Total lifetime reproductive success (mean ± SD) |  |
| --- | --- | --- |
| Low | 846 (225.8 ± 55.36) |  |
| Mean | 1248 (241.8 ± 40.88) |  |
| High | 1295 (243.7 ± 47.96) |  |
| Unpredictable | 1410 (248.3 ± 47.73) |  |
|  | | |
| F0 treatment | F1 treatment | Total lifetime reproductive success (mean ± SD) |
| Low | Low | 7600 (217.1 ± 113.8) |
|  | Mean | 7877 (224.6 ± 109.3) |
|  | High | 5697 (164.0 ± 91.98) |
|  | Unpredictable | 6875 (193.9 ± 110.1) |
|  |  |  |
| Mean | Low | 10520 (250.4 ± 99.12) |
|  | Mean | 10807 (251.0 ± 110.3) |
|  | High | 8137 (189.0 ± 65.37) |
|  | Unpredictable | 9351 (217.0 ± 115.8) |
|  |  |  |
| High | Low | 8503 (197.6 ± 140.3) |
|  | Mean | 10078 (245.3 ± 95.15) |
|  | High | 7322 (173.9 ± 87.53) |
|  | Unpredictable | 9615 (223.3 ± 89.91) |
|  |  |  |
| Unpredictable | Low | 8759 (259.9 ± 146.1) |
|  | Mean | 9208 (217.6 ± 106.0) |
|  | High | 6591 (177.5 ± 80.03) |
|  | Unpredictable | 7723 (202.0 ± 141.4) |
